# A Possible Therapeutic Application of the Selective Inhibitor of Urate Transporter 1, Dotinurad, for Metabolic Syndrome, Chronic Kidney Disease, and Cardiovascular Disease

**DOI:** 10.3390/cells13050450

**Published:** 2024-03-04

**Authors:** Hidekatsu Yanai, Hiroki Adachi, Mariko Hakoshima, Sakura Iida, Hisayuki Katsuyama

**Affiliations:** Department of Diabetes, Endocrinology and Metabolism, National Center for Global Health and Medicine Kohnodai Hospital, 1-7-1 Kohnodai, Ichikawa 272-8516, Chiba, Japan; dadachidm@hospk.ncgm.go.jp (H.A.); d-hakoshima@hospk.ncgm.go.jp (M.H.); d-20iida@hospk.ncgm.go.jp (S.I.); d-katsuyama@hospk.ncgm.go.jp (H.K.)

**Keywords:** ATP-binding cassette transporter G2, chronic kidney disease, dotinurad, hyperuricemia, organic anion transporter1/3, urate transporter 1

## Abstract

The reabsorption of uric acid (UA) is mainly mediated by urate transporter 1 (URAT1) and glucose transporter 9 (GLUT9) in the kidneys. Dotinurad inhibits URAT1 but does not inhibit other UA transporters, such as GLUT9, ATP-binding cassette transporter G2 (ABCG2), and organic anion transporter 1/3 (OAT1/3). We found that dotinurad ameliorated the metabolic parameters and renal function in hyperuricemic patients. We consider the significance of the highly selective inhibition of URAT1 by dotinurad for metabolic syndrome, chronic kidney disease (CKD), and cardiovascular disease (CVD). The selective inhibition of URAT1 by dotinurad increases urinary UA in the proximal tubules, and this un-reabsorbed UA may compete with urinary glucose for GLUT9, reducing glucose reabsorption. The inhibition by dotinurad of UA entry via URAT1 into the liver and adipose tissues increased energy expenditure and decreased lipid synthesis and inflammation in rats. Such effects may improve metabolic parameters. CKD patients accumulate uremic toxins, including indoxyl sulfate (IS), in the body. ABCG2 regulates the renal and intestinal excretion of IS, which strongly affects CKD. OAT1/3 inhibitors suppress IS uptake into the kidneys, thereby increasing plasma IS, which produces oxidative stress and induces vascular endothelial dysfunction in CKD patients. The highly selective inhibition of URAT1 by dotinurad may be beneficial for metabolic syndrome, CKD, and CVD.

## 1. Introduction

**Figure 1:**
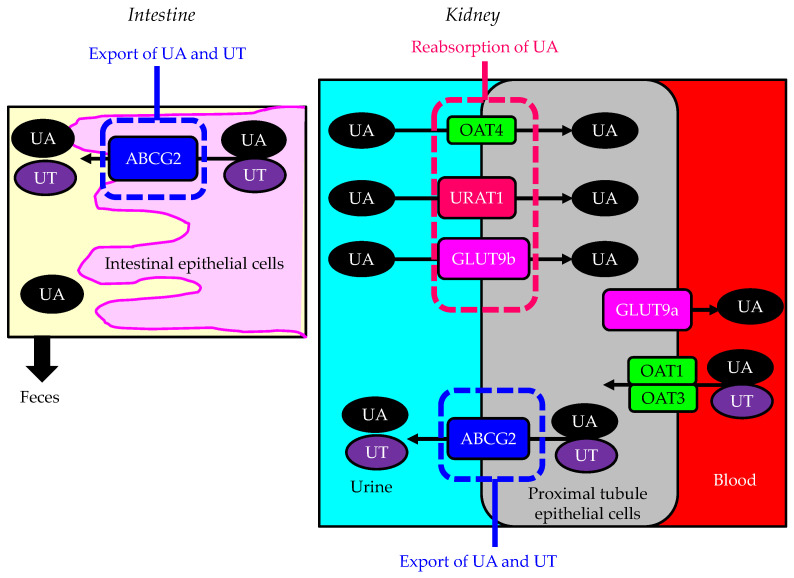
Urate transporters in the kidneys and intestine. Black arrows indicate the flow of uric acid and uremic toxins. ABCG2—ATP-binding cassette transporter G2; GLUT9—glucose transporter 9; OAT—organic anion transporter; UA—uric acid; URAT1—urate transporter 1; UT—uremic toxin.

Urate transporter 1 (URAT1), which is a urate anion exchanger that regulates serum uric acid (UA) levels in the human kidney, was identified in 2002 [1], and it has been targeted by uricosuric agents. In humans, renal reabsorption of UA into the blood plays an important role in controlling serum UA levels. The UA exchange is mediated by various molecules expressed in the renal proximal tubule [2,3] (Figure 1). UA enters the proximal tubule epithelial cells in exchange for monocarboxylate via apical URAT1 and for dicarboxylate via the apical organic anion transporter (OAT) 4 [4]. OAT1 and OAT3 on the basolateral membrane of epithelial cells transport UA from the renal interstitial into the renal proximal tubule epithelial cells [4,5]. Renal UA reabsorption is mainly mediated by URAT1 and glucose transporter 9 (GLUT9) [1,6,7,8]. Apical GLUT9b plays a significant role in UA reabsorption; the reabsorbed UA exits the proximal tubule epithelial cells into the blood through basolateral GLUT9a [4]. The ATP-binding cassette transporter G2 (ABCG2) has been identified as a high-capacity UA exporter that mediates renal and/or extra-renal (intestinal) UA excretion [9,10].

Uricosuric agents have been developed to target such UA transporters and have been used as therapeutic agents for hyperuricemia. Probenecid inhibits URAT1 and GLUT9 [11]. Benzbromarone also inhibits URAT 1 and GLUT 9 [12]. Lesinurad and arhalofenate inhibit URAT1 and OAT4 [11]. It has been difficult to accurately evaluate the function of URAT1 because the previous uricosuric agents inhibited not only URAT1 but also GLUT9 and OAT4.

A highly selective inhibitor of URAT1, dotinurad, was developed [13] and is available in Japan. Unexpectedly, we found that dotinurad improved serum lipids, blood pressure, body weight, albuminuria, and the estimated glomerular filtration rate (eGFR); in addition, it reduced serum UA in patients with hyperuricemia complicated by CKD and diabetic kidney disease (DKD) [14]. Furthermore, the 24 week-dotinurad treatment favorably affected arterial stiffness and oxidative stress, suggesting that dotinurad provides off-target vascular protection [15]. 

Dotinurad is characterized by its high selectivity, as it inhibits URAT1 but does not inhibit other UA transporters, such as ABCG2 and OATs. Here, we discuss the influences of the inhibition of URAT1 and the non-inhibition of other UA transporters on metabolic syndrome, CKD, and cardiovascular disease (CVD). Therefore, the effects of dotinurad beyond UA lowering are also the subject of discussion.

## 2. The Association of URAT1 and Other UA Transporters with Metabolic Syndrome

### 2.1. Metabolic Syndrome and Hyperuricemia 

Hyperuricemia is significantly associated with the development and severity of metabolic syndrome. A meta-analysis showed that higher serum UA levels led to an increased risk of metabolic syndrome, with a linear dose–response relationship [16]. The serum UA concentrations increased with the number of components of metabolic syndrome adjusted for age, sex, creatinine clearance, and alcohol, and diuretic use [17]. Multivariate analyses showed that the visceral fat area (VFA) was the most important determinant of elevation in serum UA and a decrease in UA clearance [18]. The magnitude of the insulin resistance and the serum UA levels were significantly related; insulin resistance was also significantly and inversely related to urinary UA clearance, and urinary UA clearance was significantly and inversely associated with serum UA levels [19]. Insulin resistance due to visceral fat accumulation may increase serum UA by decreasing renal UA clearance in patients with metabolic syndrome.

### 2.2. The Effect of Insulin Resistance on URAT1 Expression 

To elucidate the mechanism of obesity and metabolic syndrome-related hyperuricemia, the expression of URAT1 was investigated [20]. The protein level of URAT1 increased in the kidneys of leptin-deficient mice (ob/ob mice) [20]. Furthermore, the quick fat diet (crude fat content: 13.6%) enhanced the protein level of URAT1 in the kidneys of C57BL/6 mice [20]. Insulin-resistant Otsuka Long–Evans Tokushima Fatty (OLETF) rats and the control, Long–Evans Tokushima Ohtsuka (LETO) rats, were used as a model for acute hyperuricemia [21]. The OLETF rats showed a significantly higher incidence of hyperuricemia compared to the control LETO rats, indicating that insulin resistance induces hyperuricemia following a high-purine load [21]. Following a high-purine load, insulin resistance enhanced UA reabsorption through upregulation of the URAT1 expression [21].

A high-fructose diet (HFD) upregulated the expression of GLUT9 and URAT1 in the kidneys and increased the serum UA concentration in rats [22]. Another study also revealed that long-term HFD significantly upregulated the protein expression of GLUT9 and URAT1 in the kidneys of mice [23]. Resveratrol is a polyphenol that is abundant in plants; it has been reported to exert anti-inflammatory and antioxidative effects, inhibit lipid peroxidation, and extend life in mice [24]. Furthermore, the effects of resveratrol on the amelioration of insulin resistance and liver and kidney pathologies have been shown in several animal models [25,26]. Compared with those in the HFD group, the protein expression levels of GLUT9 and URAT1 were significantly lower in the HFD group treated with resveratrol. Insulin resistance enhanced the expression of URAT1 and GLUT9. 

### 2.3. The Effect of Insulin on UA Transport by Other Urate Transporters 

Insulin and hyperinsulinemia reduce the renal fractional excretion of UA and play a key role in the genesis of hyperuricemia and gout. Physiological euglycemic hyperinsulinemia induced by insulin infusion in healthy volunteers acutely reduced urinary UA, suggesting a significant contribution of insulin to the pathogenesis of hyperuricemia [27,28,29]. In rats, insulin decreased urinary UA excretion, with a concurrent increased expression of URAT1 and a decreased expression of ABCG2 [30]. There was an increased expression of GLUT9 in the kidneys of streptozotocin-induced diabetic mice [31]. The heterologous expression of individual UA transporters in Xenopus oocytes revealed that insulin increased UA transport by GLUT9, OAT1, and OAT3 and decreased UA transport by ABCG2 [32].

**Figure 2:**
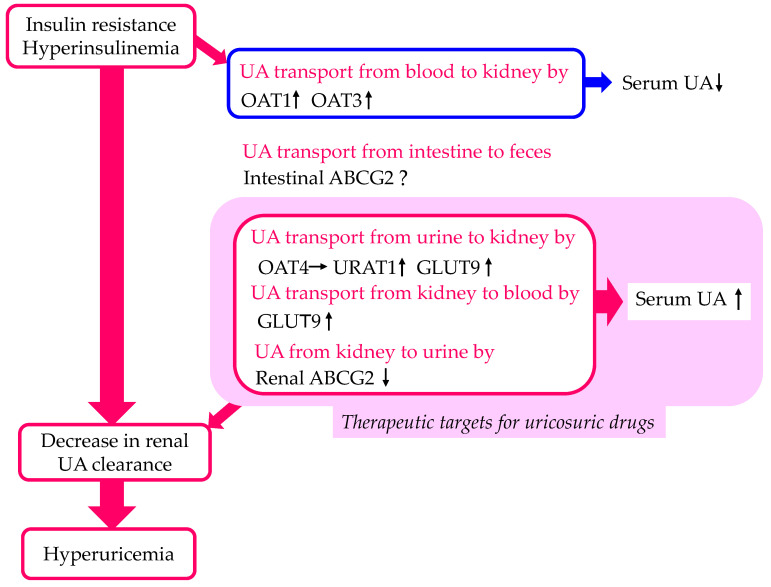
Changes in UA transport by UA transporters in the kidneys and intestine by insulin resistance and hyperinsulinemia. Upward- and downward-facing arrows indicate increase or decrease in substances or expression of molecules, respectively. Right arrow and ? indicate no change and no available data about change of substances or expression of molecules, respectively. ABCG2—ATP-binding cassette transporter G2; GLUT9—glucose transporter 9; OAT—organic anion transporter; UA—uric acid; URAT1—urate transporter 1.

The effects of insulin resistance and hyperinsulinemia on UA transport by each of the UA transporters are shown in Figure 2. Insulin resistance and hyperinsulinemia increase UA transport by URAT1 and GLUT9 and decrease UA transport by ABCG2, which may induce a decrease in renal UA clearance. Therefore, URAT1, GLUT9, and ABCG2 can be therapeutic targets for uricosuric drugs in patients with insulin resistance and hyperinsulinemia.

### 2.4. The Effect of Inhibition of URAT1 on Metabolic Parameters in Humans

We found that dotinurad reduced body weight, blood pressure, HbA1c, serum low-density lipoprotein-cholesterol (LDL-C), triglyceride (TG), and non-high-density lipoprotein-cholesterol (non-HDL-C), as well as serum UA, in patients with CKD and DKD [14]. To our knowledge, our study is the first to report such metabolic effects of dotinurad. We speculated that dotinurad selectively inhibits URAT1 and increases the urinary concentration of UA in the proximal tubules; this un-reabsorbed UA may compete with urinary glucose for apical GLUT9b, reducing glucose reabsorption, which may induce improvements in HbA1c, serum lipids, blood pressure, and body weight.

### 2.5. The Effect of Inhibition of URAT1 on Metabolic Parameters in Mice

Tanaka, Y. et al. found that URAT1 was also expressed in the liver, white adipose tissue (WAT), and brown adipose tissue (BAT) in addition to the kidneys [33]. Dotinurad administration significantly ameliorated high-fat diet-induced obesity and insulin resistance [33]. Serum TG in high-fat diet-fed mice was elevated in comparison with that in normal-fat diet-fed mice, and dotinurad significantly reduced serum TG in both types of mice [33]. Remarkably, a high-fat diet induced nonalcoholic fatty liver disease (NAFLD), which was attenuated by dotinurad [33]. Various factors, such as pro-inflammatory cytokines released from adipose tissues, hypercholesterolemia, and hyperuricemia, contribute to the development of NAFLD in high-fat diet-induced obese mice [34]. Hyperuricemia directly induces fat accumulation and inflammation in hepatocytes through URAT1 [35]. Dotinurad may improve NAFLD by inhibiting extracellular UA uptake in hepatocytes via URAT1, resulting in a reduction in lipid deposition and inflammation. The re-browning of brown adipose tissue (BAT) and the browning of epididymal white adipose tissue (WAT) may be also associated with an improvement in NAFLD via adipokines [36].

WAT could be converted to beige adipose tissue (browning), which increases energy expenditure by activating the uncoupling protein 1 (UCP1), which improves systemic insulin resistance [36,37]. The uptake of UA in WAT by URAT1 leads to WAT dysfunction and the deterioration of systemic insulin resistance [38]. In epididymal WAT, dotinurad significantly increased the UCP1 expression under high-fat diet conditions, indicating that the selective inhibition of URAT1 led to the browning of WAT under high-fat diet conditions [33]. A previous study showed that the enhanced UA uptake into WAT via URAT1 and the elevation in the intracellular UA led to the inhibition of the leptin–AMP-activated protein kinase (AMPK) pathway, which resulted in a reduction in the UCP1 expression in WAT [37].

The upregulation of the expression and activity of UCP1 in BAT plays an important role in the improvement of glucose metabolism and insulin sensitivity [39]. The UCP1 levels in BAT were significantly increased by dotinurad [33]. The uptake of UA can increase the oxidative stress in adipocytes, which induces insulin resistance [40]. The reactive oxygen species (ROS) levels in BAT were significantly reduced by treatment with dotinurad [33].

### 2.6. The Effects of Other UA-Lowering Drugs on Metabolic Parameters

Allopurinol and febuxostat are xanthine oxidase (XO) inhibitors that reduce the hepatic production of UA. In comparison with no treatment, the allopurinol and febuxostat treatments induced a significant reduction in body weight, systolic blood pressure, blood glucose, insulin, and lipids in rat models of insulin resistance and metabolic syndrome [41]. 

Allopurinol significantly reduced hepatic steatosis, epididymal fat, serum UA, the homeostatic model assessment for insulin resistance (HOMA-IR), hepatic enzyme levels, and cholesterol in the HFD-fed OLETF rats [42]. The hepatic expression of lipogenic genes, such as sterol regulatory element-binding protein 1c (SREBP-1c) and stearoyl-CoA desaturase 1 (SCD-1), was significantly upregulated in the OLETF and the HFD-fed OLETF rats compared with the LETO rats. However, allopurinol significantly downregulated SREBP-1c and SCD-1 gene expressions in the HFD-fed OLETF rats. Peroxisome proliferator-activated receptor alpha (PPARα) and carnitine palmitoyl-transferase 1 (CPT-1) were significantly downregulated in the OLETF and the HFD-fed OLETF rats compared with the LETO rats [42]. However, allopurinol improved the downregulation of lipid oxidation genes observed in the HFD-fed OLETF rats. The hepatic mRNA expression of tumor necrosis factor-alpha (TNF-α) was significantly increased in the OLETF and the HFD-fed OLETF rats, and this increase was abolished by allopurinol. In addition, allopurinol significantly decreased endoplasmic reticulum (ER) stress-induced protein expression, in comparison with the no-treatment group.

Insulin resistance increases the expression of SREBP-1c, which increases fatty acid (FA) synthesis [43]. Hepatic FA metabolism is controlled by the combination of FA uptake, FA export by very-low-density lipoprotein (VLDL) secretion, FA synthesis by SREBP-1c, and FA oxidation by β-oxidation. The entry of FA into mitochondria depends on CPT-1. One of the major regulators of CPT-1 is PPARα [44,45,46,47]. The activation of PPARα induces the transcription of genes associated with FA oxidation [44,48,49]. SCD1 plays a crucial role in FA oxidation, FA synthesis, and storage [50]. It was proposed that SCD1 plays a crucial role in the development of obesity in Mediterranean countries [51]. In experimental animals, SCD1 was significantly associated with obesity and insulin resistance [52,53]. Therefore, the allopurinol-mediated downregulation of SREBP-1c and SCD-1 genes and the upregulation of PPARα and CPT-1 in the HFD-fed OLETF rats indicate that allopurinol has a beneficial effect on hepatic steatosis in insulin resistance.

The relationship between the decrease in serum UA and VFA reduction in patients with gout was investigated [54]. The UA-lowering therapy (ULT) (febuxostat 20–80 mg/day or benzbromarone 25–50 mg/day) resulted in a decrease in the serum UA level, accompanied by a decrease in VFA. Using the multiple regression model, change in serum UA was a significant determinant of the decrease in VFA (beta, 0.302; *p* = 0.001). The reduction in serum UA is positively associated with reduced VFA, providing a rationale for clinical trials to affirm whether ULT promotes the loss of visceral fat in patients with gout. The ULT significantly reduced body weight, blood pressure, serum TG and total cholesterol levels, aspartate aminotransferase (ALT), and aspartate aminotransferase (AST).

Treatment with the XO inhibitor, topiroxostat, suppressed weight gain compared to control without any impact on food intake in diabetic obese mice [55]. However, the weight of the fat pads and the hepatic and muscle TG content did not change. Prehypertensive, obese adolescents, aged 11 to 17 years, were randomized to the XO inhibitor, allopurinol, uricosuric, probenecid, or placebo in a randomized, double-blinded, placebo-controlled trial (RCT) [56]. The subjects treated with ULT showed a significantly high reduction in blood pressure. 

The effects of UA-lowering drugs on body weight, visceral fat, blood pressure, glucose metabolism, and hepatic steatosis are shown in Table 1. These results suggest that lowering serum UA improves metabolic parameters, regardless of whether XO inhibitors or uricosuric drugs are used.

### 2.7. The Possible Mechanisms of an Improvement in Metabolic Parameters by Dotinurad

**Figure 3:**
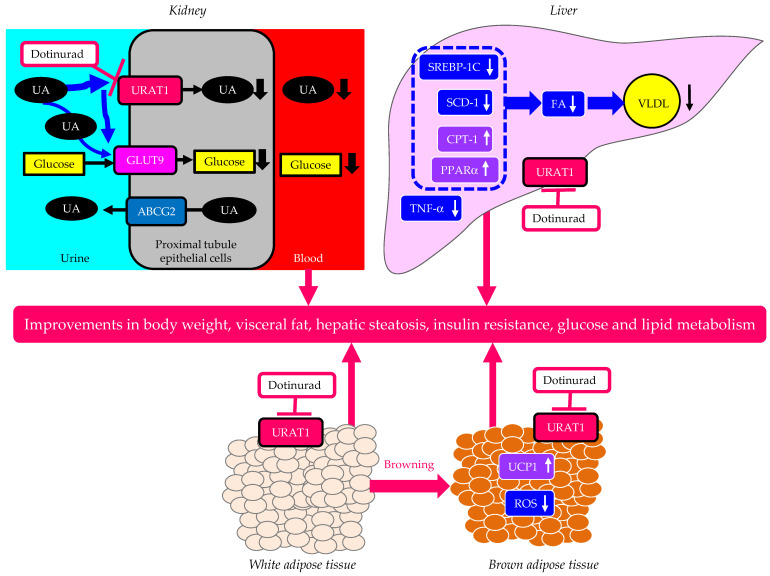
The possible mechanisms of an improvement in metabolic parameters by dotinurad. Upward- and downward-facing arrows indicate increase or decrease in substances or expression of molecules, respectively. ABCG2—ATP-binding cassette transporter G2; CPT-1—carnitine palmitoyl-transferase 1; FA—fatty acid; GLUT9—glucose transporter 9; PPARα—proliferator-activated receptor alpha; ROS—reactive oxygen species; SCD-1—stearoyl-CoA desaturase 1; SREBP-1c—sterol regulatory element-binding protein 1c; TNF-α—tumor necrosis factor-alpha; UA—uric acid; UCP1—uncoupling protein 1; URAT1—urate transporter 1; VLDL—very-low-density lipoprotein.

The possible mechanisms of an improvement in metabolic parameters by dotinurad are shown in Figure 3. In the kidney, dotinurad selectively inhibits URAT1 and increases the urinary concentration of UA in the proximal tubules; this un-reabsorbed UA may compete with urinary glucose for apical GLUT9b, reducing glucose reabsorption, which may induce an improvement in HbA1c, serum lipids, blood pressure, and body weight. In the liver, the inhibition by dotinurad of UA entry into the liver via URAT1 may upregulate the genes associated with FA oxidation and may downregulate the genes associated with FA synthesis and inflammation, which improve hepatic steatosis, systemic insulin resistance, and serum lipids. The inhibition of URAT1 in WAT by dotinurad induces the browning of WAT, and the inhibition of URAT1 in BAT increases the expression of UCP-1 and decreases the production of ROS, which may reduce body weight and visceral fat and may improve insulin resistance as well as glucose and lipid metabolism.

## 3. The Association of URAT1 and Other UA Transporters with CKD

### 3.1. CKD and Hyperuricemia 

UA induces hypertension through its effects on endothelial function and impaired nitric oxide (NO) production [57]. Hypertension can be the initial trigger leading to renal damage [58]. Hyperuricemia is caused by the activation of vasoactive and inflammatory processes [59], which may induce CKD. Histologic analyses showed the presence of arteriolosclerosis and tubulointerstitial injury in hyperuricemia-induced renal damage [60]. Serum UA levels were significantly correlated with vascular resistance at both the afferent and efferent arteriole in the glomerulus, suggesting that hyperuricemia may be harmfully associated with glomerular perfusion [61]. Furthermore, the activation of the renin–angiotensin system (RAS) by hyperuricemia may be associated with the development of CKD [62]. The activation of RAS can induce renal vasoconstriction and reduce renal plasma flow. UA may also increase oxidative stress and pro-inflammatory cytokines and induce the proliferation of vascular smooth muscle cells (SMC) [2]. UA crystals can cause tubular damage through inflammation mediated by crystals [2]. 

High serum UA levels are significantly associated with an increased risk of CKD. A total of 2059 community-dwelling Japanese subjects aged ≥40 years without CKD were followed for 5 years [63]. CKD increased with higher serum UA levels, with 21% (serum UA 4.1–4.9 mg/dL), 47% (serum UA 5.0–5.8 mg/dL), and 210% (serum UA ≥ 5.9 mg/dL). Similarly, there were positive associations between the serum UA level and the adjusted risk of developing a decline in eGFR < 60 mL/min/1.73 m^2^ [63]. This study showed that hyperuricemia is a significant risk factor for a decline in eGFR and albuminuria. A screened cohort study including 48,177 individuals showed that the calculated incidences of end-stage renal disease (ESRD) per 1000 people increased from 1.22 (without hyperuricemia) to 4.64 with hyperuricemia for men, and also increased from 0.87 (without hyperuricemia) to 9.03 with hyperuricemia for women [64]. Hyperuricemia is significantly associated with the development and progression of CKD.

### 3.2. The Effect of CKD on Renal URAT1 Expression 

The possibility that the hyperuricemia observed in renal dysfunction was due to decreased UA clearance from the kidneys due to decreased renal function has previously been considered. As various UA transporters exist in the proximal tubule of the kidney, the influence of CKD progression on these transporters must be considered. Both the mRNA expression and the immunohistochemistry of the URAT1 were decreased in the CKD rat model [65].

### 3.3. The Effect of CKD on Other Urate Transporter Expressions

Both the mRNA expression and the immunohistochemistry of GLUT9 and ABCG2 in the kidneys were decreased in the CKD rat model [65]. CKD patients accumulate uremic toxins (UTs) in the body and potentially require dialysis. ABCG2 is a major transporter of UTs such as indoxyl sulfate (IS) [66]. ABCG2 regulates the renal and intestinal excretion of IS and strongly affects CKD survival rates [67]. Considering the decreased renal clearance of UA and UT in CKD rat models, intestinal ABCG2 may play a compensatory role [67].

OAT1/3-mediated active tubular secretory clearance was reduced by 50% relative to the GFR decline in severe CKD, whereas the change in the active secretion in mild and moderate CKD was proportional to GFR [68]. The 4-pyridoxic acid (PDA) was the biomarker used to evaluate the inhibition of OAT1 and OAT3 [69,70,71]. Recent clinical studies have reported an increase in plasma PDA in CKD populations [72,73]. The changes in plasma PDA concentrations in CKD exceeded those reported after probenecid inhibition and were likely a reflection of deteriorating active renal secretion. OAT1 and OAT3 play a key role in the handling of UTs such as IS [74]. UTs inhibit OAT1 and OAT3, which contribute to the decline in renal drug and UT clearance in patients with CKD [75].

### 3.4. The Effect of IS on CKD

The accumulation of IS has been observed in the serum of CKD patients. Dietary protein-derived tryptophan is metabolized into indole by intestinal bacteria. Indole is absorbed into the blood and is metabolized to IS in the liver [76]. IS is normally excreted into urine. In CKD, however, a reduced renal IS clearance leads to the elevation of serum IS. IS leads to progression of both tubulointerstitial fibrosis and glomerular sclerosis. Moreover, IS induces oxidative stress in tubular cells, mesangial cells, vascular SMC, and endothelial cells, which are also involved in the progression of CKD. 

Serum IS levels increased gradually with the decrease in renal function and reached the highest level in CKD stage 5 [77]. Serum IS was measured in 604 pediatric participants (mean eGFR of 27 ± 11 mL/min/1.73 m^2^) following enrolment into the prospective Cardiovascular Comorbidity in Children with CKD study [78]. During a median follow-up time of 2.2 years, the composite renal survival endpoint, defined as a 50% loss of eGFR, or eGFR < 10 mL/min/1.73 m^2^, or the start of renal replacement therapy, was investigated. The median survival time was shorter in patients with IS levels in the highest versus the lowest quartile for IS (1.5 years, 95%CI [1.1,2.0] versus 6.0 years, 95%CI [5.0,8.4]). Serum IS levels were significantly associated with renal survival, which was independent of other risk factors, such as baseline eGFR, proteinuria, and blood pressure. 

**Figure 4:**
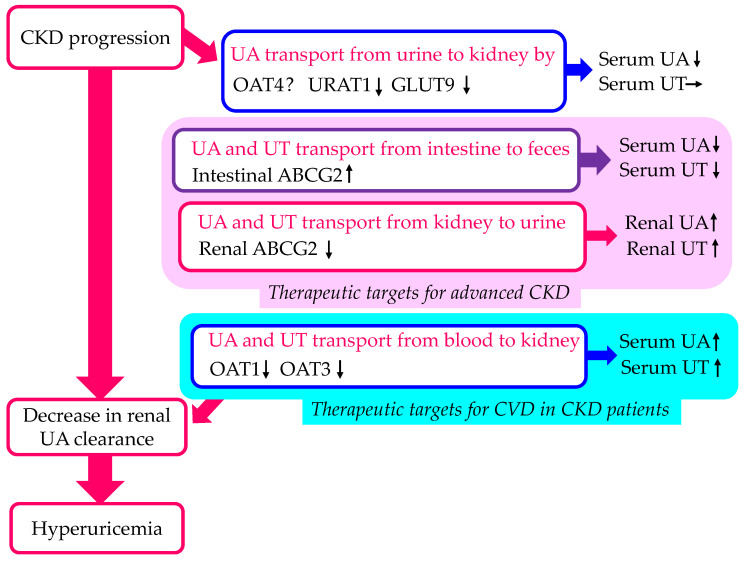
Changes in UA transport by urate transporters in the kidneys and intestine by CKD progression. Upward- and downward-facing arrows indicate increase or decrease in substances or expression of molecules, respectively. ? indicates no available data about change of substances or expression of molecules. ABCG2—ATP-binding cassette transporter G2; CKD—chronic kidney disease; CVD—cardiovascular disease; GLUT9—glucose transporter 9; OAT—organic anion transporter; UA—uric acid; URAT1—urate transporter 1.

The effects of CKD progression on UA transport by each UA transporter are shown in Figure 4. CKD progression decreases the expression of URAT1 and GLU9, which may increase serum UA, and decreases the expression of OAT1 and OAT3, which may increase serum UA and UT. Furthermore, CKD progression decreases the expression of renal ABCG2, which may increase renal UA and UT, and increases intestinal ABCG2, which may reduce serum UA and UT. To suppress the progression of CKD, UT should be smoothly excreted from the body. For this purpose, drugs that do not inhibit ABCG2 are desired.

### 3.5. The Effect of Inhibition of URAT1 on CKD

The effects of UA-lowering drugs on renal function and renal outcome are shown in Table 2.

We previously reported that starting treatment with dotinurad decreased blood urea nitrogen (BUN) and increased eGFR, and the increased dose of dotinurad further decreased BUN and increased eGFR with a reduction in UA in a diabetic patient with CKD stage G4 [79]. In this case, an improvement in albuminuria after the start of dotinurad was also observed [79]. In our study, the 6-month dotinurad treatment improved albuminuria and eGFR in hyperuricemic patients [14]. In another study, although eGFR did not significantly change in patients whose eGFR was 30 or more, dotinurad significantly improved the eGFR in patients whose eGFR was less than 30 [80]. The frequency of patients with improved eGFR was significantly higher in patients whose eGFR was less than 30 (*p* = 0.038) than in patients whose eGFR was 30 or more. In the multivariate logistic regression analysis, the baseline eGFR < 30 and the achievement of a serum UA level of ≤ 6.0 mg/dL were significantly associated with improved eGFR (*p* = 0.033 and *p* = 0.015, respectively) [80]. This study suggested that dotinurad may have the potential to improve renal function in patients with advanced CKD. 

Yanai, K. et al. investigated the efficacy and safety of dotinurad in 34 hyperuricemic patients with advanced CKD (stages G3–5) [81]. The 12-month dotinurad treatment significantly reduced the annual decline in eGFR from −6.0 ± 12.9 to −0.9 ± 4.6 mL/min/1.73 m^2^/year (*p* < 0.05), but such a change was not observed in the control group, suggesting that dotinurad can attenuate renal function decline in advanced CKD individuals with hyperuricemia.

### 3.6. The Effects of Other UA-Lowering Drugs on CKD

In an RCT of 54 hyperuricemic patients with CKD, the patients were randomly assigned to treatment with allopurinol or to continuation of the usual therapy for 12 months [82]. The serum creatinine level in the allopurinol group tended to be lower than that in the controls after 12 months (*p* = 0.08). The combined endpoints of significant deterioration in renal function and dialysis dependence were observed in 16% and 46.1% of the allopurinol group and control group, respectively (*p* = 0.015).

One hundred and thirteen patients with eGFR < 60 mL/min were randomly assigned to treatment with allopurinol at 100 mg/day or to the continuation of the usual therapy [83]. In the control group, eGFR decreased by 3.3 ± 1.2 mL/min/1.73 m^2^, and in the allopurinol group, eGFR increased by 1.3 ± 1.3 mL/min/1.73 m^2^ after 24 months. The post hoc analysis of a long-term follow-up after completion of the 2-year RCT showed that during the initial and long-term follow-up (median, 84 months), the allopurinol group had a significantly lower occurrence of a renal event compared with the control group (hazard ratio [HR], 0.32; 95% confidence interval [CI], 0.15–0.69; *p* = 0.004) [84]. 

A greater reduction in serum UA with febuxostat was associated with an increase in eGFR and decreased proteinuria in patients with CKD stages 3b, 4, and 5 [85]. In a 1-year cohort study of 73 hyperuricemic patients with eGFR < 45 mL/min, the treatment in 51 patients was changed from allopurinol to febuxostat, and the other 22 patients continued treatment with allopurinol [86]. The serum UA levels significantly decreased from 6.1 ± 1.0 to 5.7 ± 1.2 mg/dl in the febuxostat group and significantly increased from 6.2 ± 1.1 to 6.6 ± 1.1 mg/dl in the allopurinol group. The eGFR decreased from 27.3 to 25.7 mL/min in the febuxostat group and from 26.1 to 19.9 mL/min in the allopurinol group, suggesting that febuxostat slowed the progression of renal disease in the CKD cohort in comparison with allopurinol. In an RCT, receiving febuxostat for 12 weeks reduced the urinary levels of fatty acid-binding protein 1 (FABP1), albumin, and β2-microglobulin, whereas the levels of these markers did not change in the control group [87]. Urinary FABP1 and β2-microglobulin are the markers for proximal tubular impairment [88,89]. However, the meta-analysis showed no significant differences in the changes in serum creatinine from the baseline between the febuxostat and allopurinol groups [90]. The eGFR did not significantly change within 3 months. A significant difference existed in the changes in albuminuria levels from the baseline between the febuxostat and allopurinol groups (mean difference [MD], −80.47 mg/gram creatinine [gCr]; 95% CI, −149.29 to −11.64 mg/gCr; *p* = 0.02) [90]. A nationwide database analysis showed that a lower risk of progression to dialysis was observed in pre-dialysis stage 5 CKD febuxostat users without compromising survival [91].

Topiroxostat treatment resulted in a significant reduction in serum UA, systolic and diastolic blood pressures, and urinary protein compared with the baseline values [92]. However, serum creatinine, urinary N-acetyl-beta-D-glucosaminidase (NAG), which is the marker for renal tubular impairment [93], and eGFR did not change significantly [92]. Another study reported that topiroxostat significantly improved eGFR and reduced the urinary albumin/creatinine ratio compared to a placebo [94].

A 13-year inception cohort study showed that compared with allopurinol, benzbromarone therapy was associated with a reduced risk of progression to dialysis; the adjusted HR was 0.50 (95% CI, 0.25–0.99) [95]. We could not find any RCTs and meta-analyses that investigated the effect of probenecid on CKD.

### 3.7. The Effects of Febuxostat and Dotinurad on Advanced CKD

Serum IS levels increased gradually with the decrease in renal function and reached the highest level at CKD stage 5 [77]. The serum IS concentration is significantly associated with renal survival [78]. Therefore, ABCG2-mediated excretion of IS [66] may be more critical for patients with CKD stage 4 or 5. The start of dotinurad, which did not inhibit ABCG2, improved eGFR in our patients with CKD stage 4 [79]. In this case, an improvement in albuminuria after the start of dotinurad was also observed [79]. Although eGFR did not significantly change in patients with 30 ≤ eGFR < 45 and eGFR ≥ 45, dotinurad significantly improved eGFR in patients with eGFR < 30 [80]. However, in a cohort study of 778 gout patients, febuxostat reduced eGFR (19.1 mL/min/1.73 m^2^ at baseline) by 0.7 mL/min/1.73 m^2^ in patients with CKD stage 4, 5 [96]. Another study also showed that the 12-month febuxostat treatment did not significantly improve the eGFR in patients with CKD stage 4, 5 (*p* = 0.13) [97]. 

**Table 2:** The effects of UA-lowering drugs on renal function and renal outcome.

UA-Lowering Drugs	XO Inhibitors			Uricosuric Drugs		
	Allopurinol	Febuxostat	Topiroxostat	Benzbromarone	Probenecid	Dotinurad
**Inhibition of UA** **Transporters**		**ABCG2**	**ABCG2**	**ABCG2**	**ABCG2**	**URAT1**
				**URAT1**	**URAT1**	
				**GLUT9**	**GLUT9**	
				**OAT1**	**OAT1**	
				**OAT3**	**OAT3**	
Albuminuria	No data	Improved [85,87,90]	Improved [92]	No data	No data	Improved [14,79]
eGFR or serum creatinine	Improved [82,83]	Improved [85]	Not improved [93] and Improved [94]	No data	No data	Improved [14,79,80,81]
eGFR in patients with CKD stage 4 and 5	No data	Not improved [96,97]	No data	No data	No data	Improved [79,80,81]
Proximal tubular impairment	No data	Improved [87]	Not improved [93]	No data	No data	No data
Renal outcomes	Improved [84]	Improved [91]	No data	Improved [95]	No data	No data

ABCG2—ATP-binding cassette transporter G2; GLUT9—glucose transporter 9; OAT—organic anion transporter; UA—uric acid; URAT1—urate transporter 1; XO—xanthin oxidase.

To suppress the progression of CKD, drugs that do not inhibit ABCG2, which excretes UTs such as IS, are desired. Febuxostat has been reported to be a strong ABCG2 inhibitor [98], and dotinurad does not inhibit ABCG2. Taniguchi, T. et al. evaluated whether hypouricemic agents, including dotinurad, affect IS clearance in rats [99]. Febuxostat caused highly significant renal IS accumulation by suppressing its excretion. Dotinurad did not significantly affect the clearance of IS. 

## 4. The Association of URAT1 and Other UA Transporters with the Development of CVD

### 4.1. The Association of URAT1 with Atherogenesis

High levels of UA are associated with the development of CVD. The plasma membrane enzyme ectonucleotide pyrophosphatase/phosphodiesterase 1 (ENPP1) was shown to inhibit the insulin receptor function, and high expression levels of ENPP1 were observed in the cells of insulin-resistant subjects [100]. Cultures of human umbilical vein endothelial cells were stimulated with insulin, UA, and the URAT1 inhibitor probenecid [101]. UA inhibited the insulin-induced Akt/endothelial nitric oxide synthase (eNOS) axis [101], suggesting that UA has a key role in reducing Akt–eNOS axis activity, which induces endothelial dysfunction [101]. UA induced ENPP1 binding to the insulin receptor, leading to an impairment of insulin signaling. Probenecid reverted such UA effects, indicating that UA intracellular uptake by URAT1 is required for its action.

The expression of URAT1 on human aortic vascular SMC was reported [102]. URAT1 was expressed in the cell membrane, and UA enters human vascular SMC via URAT1 [103]. UA upregulated C-reactive protein (CRP) mRNA in human vascular SMC (HVSMC) and human umbilical vein endothelial cells (HUVEC) [104]. UA stimulated HVSMC proliferation, whereas UA inhibited the serum-induced proliferation of HUVEC, which was attenuated by co-incubation with probenecid. UA also increased HVSMC migration and inhibited HUVEC migration. In HUVEC, UA reduced NO release. The entry of UA into cells via URAT1 may induce endothelial dysfunction and the proliferation of SMC by inducing inflammation.

### 4.2. The Association of Other UA Transporters with Atherogenesis

NOD-, LRR-, and pyrin domain-containing protein 3 (NLRP3) is an intracellular sensor that detects microbial motifs, endogenous danger signals, and environmental irritants, resulting in the formation and activation of the NLRP3 inflammasome. The assembly of the NLRP3 inflammasome leads to the caspase 1-dependent release of the pro-inflammatory cytokines interleukin (IL)-1β and IL-18 [105]. Soluble UA absorbed by cells through UA transporters accumulates intracellularly and activates the NLRP3 inflammasome, thereby increasing IL-1β secretion. ABCG2 excludes intracellular UA. GLUT9 and ABCG2 were expressed in macrophage-like J774.1 cells; however, URAT1 was not expressed in these cells. The entry of soluble UA via GLUT9 increased the mRNA and protein levels of ABCG2 in macrophage-like J774.1 cells, and an ABCG2 inhibitor, febuxostat, but not dotinurad, increased IL-1β production in cells pretreated with UA, suggesting that the inhibition of ABCG2 enhances IL-1β production, especially under hyperuricemic conditions, by increasing intracellular UA accumulation in macrophage-like cells [106].

### 4.3. The Effect of Inhibition of URAT1 on Atherosclerosis

The cardio-ankle vascular index (CAVI), a marker of arterial stiffness, was developed in 2004 [107]. Several studies have demonstrated that the CAVI is high in patients with various atherosclerotic risk factors and that the treatments of cardiovascular risk factors improve the CAVI [107]. A multicenter prospective cohort study with a 5-year follow-up period that included patients (aged 40–74 years) with CVD risks was performed [108]. The CAVI predicted the primary outcome (HR, 1.38; 95% CI, 1.16–1.65; *p* < 0.001). When the CAVI was incorporated into a model with known CVD risks for predicting CV events, the global χ2 value increased, suggesting that the CAVI predicted CV events. The 24-week treatment with dotinurad significantly reduced the CAVI from 9.29 to 8.92 (*p* = 0.044), suggesting that dotinurad may favorably affect arterial stiffness [15]. The derivatives of the reactive oxygen metabolite concentration at week 24 were significantly lower than those at the baseline [15]. URAT1 inhibition by dotinurad at the urate entry site on the vascular walls and the resultant attenuation of ROS production might have caused such beneficial vascular effects [103,104].

### 4.4. The Effects of Other UA-Lowering Drugs on Endothelial Function

Endothelial dysfunction is an initial phase in the atherosclerotic process. Hyperuricemia and advanced CKD, in particular, are related to endothelial dysfunction through impairment of NO bioavailability, and the markers of endothelial dysfunction are associated with the stages of CKD [109]. XO inhibitors produce benefits related to endothelial function by reducing oxidative stress [110]. A meta-analysis of RCTs showed that allopurinol therapy is associated with significantly improved endothelial function in subjects at risk of CVD, and the beneficial effects of allopurinol seemed to be more remarkable in patients with normal UA at the baseline [111]. Allopurinol has an antioxidant property, which may be associated with an improvement in endothelial function [110].

The elevated expression of the eNOS inhibitor, asymmetric dimethylarginine (ADMA), is associated with endothelial dysfunction [112,113,114,115]. Furthermore, the elevation of ADMA is associated with an increased risk of CVD. The 8-week febuxostat treatment did not show improvements in serum ADMA, high-sensitivity CRP, or vascular stiffness measured using the ankle–brachial index in patients with CKD [116]. The febuxostat treatment did not alter endothelial function, which was assessed using flow-mediated dilation during a 2-year study period in patients with asymptomatic hyperuricemia [117]. Furthermore, an RCT showed that neither topiroxostat nor febuxostat had any significant effects on arterial stiffness measured with the CAVI over 24 of weeks treatment [118].

Nakata, T. et al. compared the effects of benzbromarone and febuxostat on endothelial function in a randomized, cross-over, open-label study. Thirty patients with hyperuricemia were divided into two groups; they were initially treated with benzbromarone or febuxostat for three months; these were then switched for the next three months [119]. Endothelial function was defined by reactive hyperemia indexes (RHI), determined using Endo-PAT 2000. Adiponectin and the RHI significantly increased after treatment with benzbromarone. The changes in the RHI (*p* = 0.026) and adiponectin levels (*p* = 0.001) were significantly greater in patients treated with benzbromarone than in those treated with febuxostat. In addition to reducing UA, benzbromarone increased adiponectin and might be more beneficial for endothelial function than febuxostat.

### 4.5. The Effects of UA-Lowering Drugs on CVD

A meta-analysis was conducted to determine the association between two ULTs commonly used in clinical practice (febuxostat vs. allopurinol) with major adverse cardiac events (MACE), using 10 RCTs [120]. No significant association of either of the ULTs with all-cause mortality, myocardial infarction, or stroke was noted. The retrospective cohort study used data from the Japanese healthcare record database, including 152,166 patients; it showed that ULT for patients with asymptomatic hyperuricemia did not prevent the development of CVD [121]. In the subgroup analysis, the subjects prescribed topiroxostat had a higher risk of developing CVD (HR, 1.89; 95% CI, 1.18 to 3.03; *p* = 0.01). The meta-analysis showed that in patients without atherosclerotic disease, febuxostat likely had a similar CV risk profile to allopurinol [122]. However, in patients with a history of CVD, allopurinol treatment was associated with less CV mortality compared with febuxostat treatment.

In a large population-based cohort of gout patients, allopurinol was associated with an increased risk of composite CV events and all-cause mortality compared with benzbromarone [123]. In a large cohort of 38,888 elderly gout patients, treatment with probenecid appeared to be associated with a modestly decreased risk of CV events compared with allopurinol [124].

IS accumulates in the bodies of CKD patients. In the renal proximal tubules, IS excretion is mediated by OAT1/3 and ABCG2 (Figure 1 and Figure 4). OAT1 and OAT3 are inhibited by probenecid and benzbromarone. OAT inhibitors, such as probenecid, suppress IS uptake into the kidney, leading to increased plasma IS concentration, which is harmful for CVD in CKD patients [99]. Therefore, hypouricemic agents that do not affect OATs and ABCG2 are effective therapeutic options for the treatment of hyperuricemia complicated by CKD.

CVD can explain a large part of the high mortality observed in CKD. Elevated serum IS induces vascular alterations. In a cohort of CKD patients, the highest serum IS tertile was a powerful predictor of overall and CV mortality (*p* = 0.001 and *p* = 0.012, respectively) [125]. This indicates that serum IS may have a significant role to play in the development of CVD and higher mortality in CKD patients. 

## 5. A Summary of Unfavorable Effects of the Inhibition of ABCG2, OAT1, and OAT3 on the Kidneys and Vascular Endothelial Cells in CKD Patients

**Figure 5:**
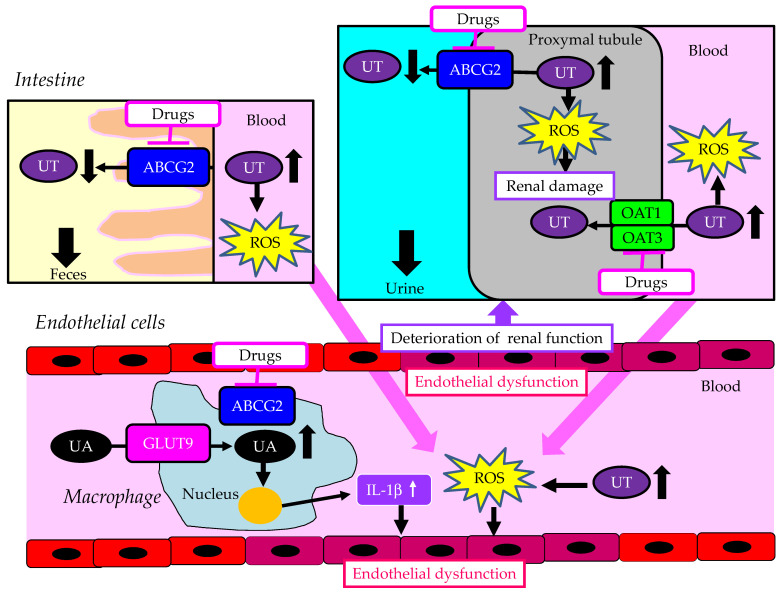
A summary of unfavorable effects of the inhibition of ABCG2, OAT1, and OAT3 on the kidneys and vascular endothelial cells in CKD patients. Upward- and downward-facing arrows indicate increase or decrease in substances. ABCG2—ATP-binding cassette transporter G2; GLUT9—glucose transporter 9; IL-1β—interleukin-1b; OAT—organic anion transporter; ROS—reactive oxygen species; UA—uric acid; UT—uremic toxin.

A summary of the unfavorable effects of the inhibition of ABCG2 and of OAT1 and OAT3 on the kidneys and vascular endothelial cells in CKD patients is shown in Figure 5. 

Among CKD patients, the inhibition of renal ABCG2 may increase renal UT accumulation, which produces ROS, resulting in renal damage. The inhibition of intestinal ABCG2 and renal OAT1/3 increases plasma UT, which produces ROS, inducing endothelial dysfunction. Endothelial dysfunction causes renal dysfunction. The inhibition of ABCG2 induces UA accumulation in macrophages due to reduced excretion of UA by ABCG2, which induces the increased secretion of IL-1b. Such inflammatory cytokines induce endothelial dysfunction. 

## 6. A Summary of the Beneficial Effects of Dotinurad on the Kidneys and Atherosclerosis in CKD Patients

**Figure 6:**
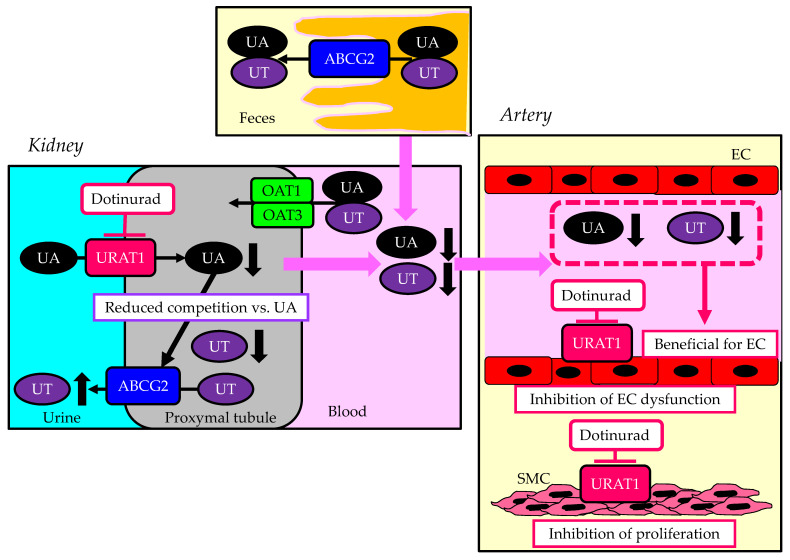
A summary of beneficial effects of dotinurad on the kidneys and atherosclerosis in CKD patients. Upward- and downward-facing black arrows indicate increase or decrease in substances. ABCG2—ATP-binding cassette transporter G2; EC—endothelial cells; OAT—organic anion transporter; ROS—reactive oxygen species; SMC—smooth muscle cells; UA—uric acid; URAT1—urate transporter 1; UT—uremic toxin.

A summary of the beneficial effects of dotinurad on the kidneys and atherosclerosis in CKD patients is shown in Figure 6. Dotinurad does not inhibit intestinal ABCG2 and renal OAT1 and OAT3, which do not increase plasma UA and UT. This property is beneficial for endothelial function. URAT1 inhibition in endothelial cells and vascular SMC by dotinurad may prevent the development and progression of atherosclerosis. In the kidneys, dotinurad reduces renal UA accumulation by inhibiting UA reabsorption, which may increase the excretion of UT into urine due to reduced competition against UA for ABCG2. This property is beneficial for renal function in CKD patients.

## 7. Possible Beneficial Effects of Dotinurad for Heart Failure (HF) 

A cohort study, which included 1665 adults aged ≥65 years, from the National Nutrition and Health Survey in elderly people in Taiwan showed that hyperuricemia was associated with HF hospitalization [126]. The National Health and Nutrition Examination Survey in China also reported that patients with hyperuricemia or gout were more likely to have HF compared with those without hyperuricemia or gout [127]. The serum UA level was reported to be an important marker of comorbidities and functional status in patients with HF with a preserved ejection fraction (HFpEF) [128]. Hyperuricemia was an independent predictor of all-cause mortality in patients with chronic HF (CHF) [129]. In elderly multimorbid patients, acute HF prognosis appeared to be influenced by hyperuricemia independently of renal function [130]. After adjusting the confounding factors using propensity score matching, hyperuricemia was found to be a determinant of HF with a reduced ejection fraction (HFrEF) (odds ratio [OR], 1.247; 95% CI, 1.172–1.328; *p* < 0.001) [131]. Hyperuricemia significantly increased all-cause death by 2.4 times and HF readmission by 1.8 times in HFrEF patients [131]. Hyperuricemia and CKD, both individually and cumulatively, are associated with an increased mortality risk in patients with CHF [132]. The serum UA level and hyperuricemia were shown to be associated with HF readmission in an observational study in China [133]. The systematic review showed that serum UA elevation was associated with the severity and complications of congestive HF [134]. It was speculated that serum UA served as a useful surrogate marker of oxidative stress in congestive HF patients [135]. Such accumulated evidence has shown a significant association between hyperuricemia and the development and progression of HF.

The UA level at the baseline was negatively correlated with the left ventricular ejection fraction [LVEF] of the follow-ups (r = −0.19; *p* = 0.046) [131]. Elevated UA was associated with greater hemodynamic impairment in advanced HF [136]. Elevated serum UA was closely associated with right ventricular dysfunction in patients with HFpEF [137]. In patients with CHF, those with hyperuricemia had significantly lower LVEF (38.2 ± 7.0 and 44.5 ± 5.1, respectively; *p* < 0.05). Patients with hyperuricemia had a significantly thicker interventricular septum (IVS) than those without it (10.49 ± 2.9 vs. 10.93 ±1.64 mm, respectively; *p* < 0.006). The LV mass index was higher in patients with hyperuricemia (*p* < 0.001) [138]. Hyperuricemia is associated with right and left ventricular ejection dysfunction and the remodeling of the myocardium. 

The sodium–glucose cotransporter-2 inhibitor empagliflozin decreases the risk of CV death or hospitalization for HF in patients with HFrEF. An interaction between serum UA and the treatment effect suggested a benefit of empagliflozin in terms of mortality (cardiovascular and all-cause mortality) in patients with elevated serum UA (*p* for interaction = 0.005 and = 0.011, respectively) [139]. Long-term febuxostat treatment was associated with protective effects in terms of LV hypertrophy (LVH) or LV diastolic dysfunction in patients with hypertensive LVH and asymptomatic hyperuricemia. Febuxostat also displayed a trend of a reduced risk of new-onset HFpEF in patients with LVH and asymptomatic hyperuricemia [140]. After a median follow-up of 23.5 months, the primary endpoint reflected by E/e’, which is the marker for diastolic dysfunction, in the benzbromarone (URAT1 inhibitor) group reached a significant improvement when compared with the control group (*p* < 0.001) [141]. The favorable trend of freedom from the composite endpoints or new-onset HFpEF was observed in the benzbromarone group (*p* = 0.037 and *p* = 0.054, respectively) [141].

Serum UA was reported to activate NLRP3 inflammasome in cardiomyocytes, which may provide one therapeutic strategy for myocardial damage induced by serum UA [142]. UA induced myocardial hypertrophy by activating autophagy via the adenosine monophosphate kinase (AMPK)-unc-51-like kinase (ULK1) signaling pathway [143]. UA promoted cardiomyocyte injury through activation of the NLRP3 inflammasome and ROS/transient receptor potential melastatin 2 (TRPM2) channel/Ca^2+^ pathway in a myocardial infarction animal model [144]. High UA levels stress cardiomyocytes by accelerating the arginine metabolism via the upregulation of ornithine decarboxylase [145]. Recently, various direct unfavorable effects of UA on cardiomyocytes have been reported. 

Very recently, URAT1 was found to be expressed in cardiomyocytes and indeed worked as a UA transporter [146]. Dotinurad substantially attenuated high-fat diet-induced cardiac fibrosis, inflammatory responses, and cardiac dysfunction. Dotinurad could be a promising candidate as a therapeutic tool for HF. 

## 8. Conclusions

**Figure 7:**
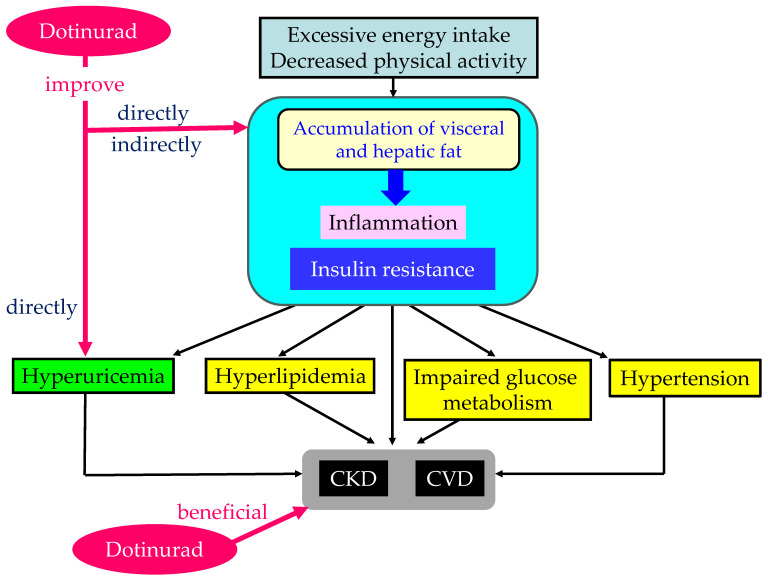
The pathophysiology of metabolic syndrome and effects of dotinurad. Red lines indicate effects of dotinurad. CKD—chronic kidney disease; CVD—cardiovascular disease.

The pathophysiology of metabolic syndrome and the effects of dotinurad are shown in Figure 7. In metabolic syndrome, excessive energy intake and decreased physical activity induce the accumulation of visceral and hepatic fat, which results in inflammation and insulin resistance. Such metabolic disturbance induces dyslipidemia, impaired glucose metabolism, hypertension, and hyperuricemia, which may lead to CKD and CVD. Dotinurad reduces serum UA and the entry of UA into adipose tissue and the liver by inhibiting URAT1. Therefore, dotinurad may directly and indirectly reduce the accumulation of visceral and hepatic fat, potentially leading to improvements in the inflammatory state and insulin resistance. These effects may improve hypertension and glucose and lipid metabolism, potentially benefiting the development and progression of CKD and CVD. Dotinurad also directly inhibits UA entry into vascular endothelial cells and SMC, potentially offering protective effects for the kidneys and CV systems. Non-inhibition of ABCG2 by dotinurad does not increase the accumulation of renal and plasma IS, as it is favorably associated with the development and progression of CKD and CVD. 

## Figures and Tables

**Table 1:** The effects of UA-lowering drugs on body weight, visceral fat, blood pressure, glucose and lipid metabolism, and hepatic steatosis.

UA-Lowering Drugs	XO Inhibitors			Uricosuric Drugs		
	Allopurinol	Febuxostat	Topiroxostat	Benzbromarone	Probenecid	Dotinurad
**Inhibition of UA Transporters**		**ABCG2**	**ABCG2**	**ABCG2**	**ABCG2**	**URAT1**
				**URAT1**	**URAT1**	
				**GLUT9**	**GLUT9**	
				**OAT1**	**OAT1**	
				**OAT3**	**OAT3**	
Body weight	Reduced	Reduced	Suppressed weight gain	Reduced	No data	Reduced
	(animal) [41]	(animal and human) [41,54]	(animal) [55]	(human) [54]		(animal and human) [14,33]
Visceral fat	Reduced	Reduced	No change	Reduced	No data	Reduced
	(animal) [42,56]	(human) [54]	(animal) [55]	(human) [54]		(animal) [33]
Blood pressure	Reduced	Reduced	No data	Reduced	Reduced [56] (human)	Reduced
	(animal and human) [41,56]	(animal and human) [41,54]		(human) [54]		(animal and human) [14,33]
Glucose metabolism	Improved	Improved	No data	No change	No data	Improved
	(animal) [41]	(animal) [41]		(human) [54]		(animal and human) [14,33]
Serum lipids	Improved	Improved	No data	Improved	No data	Improved
	(animal) [41]	(animal and human) [41,54]		(human) [54]		(animal and human) [14,33]
Hepatic steatosis	Improved	Improved	No change	Improved	No data	Improved
	(animal) [42]	(human) [54]	(animal) [55]	(human) [54]		(animal) [33]

ABCG2—ATP-binding cassette transporter G2; GLUT9—glucose transporter 9; OAT—organic anion transporter; UA—uric acid; URAT1—urate transporter 1; XO—xanthin oxidase.
